# Immunotherapy with corticosteroids in anti-neural autoantibody-associated cognitive impairment: Retrospective case series

**DOI:** 10.3389/fnagi.2022.856876

**Published:** 2022-09-27

**Authors:** Niels Hansen, Sina Hirschel, Kristin Rentzsch, Jens Wiltfang, Berend Malchow, Dirk Fitzner

**Affiliations:** ^1^Department of Psychiatry and Psychotherapy, University Medical Center Göttingen, Göttingen, Germany; ^2^Clinical Immunological Laboratory Prof. Dr. med. Winfried Stöcker, Lübeck, Germany; ^3^German Center of Neurodegenerative Diseases (DZNE), Göttingen, Germany; ^4^Neurosciences and Signaling Group, Department of Medical Sciences, Institute of Biomedicine (iBiMED), University of Aveiro, Aveiro, Portugal; ^5^Department of Neurology, University Medical Center Göttingen, Göttingen, Germany

**Keywords:** methylprednisolone, anti-neural autoantibody, mild cognitive impairment, immunotherapy, dementia

## Abstract

**Background:**

Anti-neural autoantibody-associated cognitive impairment is an increasing phenomenon in memory clinics deserving more attention to applying immunotherapy such as methylprednisolone to improve cognition. Our study aims to investigate the usefulness of intravenous high-dosage corticosteroids in a small cohort of patients suffering from anti-neural autoantibody-associated cognitive impairment.

**Materials and methods:**

We included in our retrospective case series seven patients presenting diverse neural autoantibodies and cognitive impairments varying from a mild impairment to dementia. We conducted neuropsychological and psychopathological investigations before and after the application of high intravenous methylprednisolone therapy over a 6-month period. Neuropsychological function was assessed by the CERAD (Consortium to Establish a Registry for Alzheimer’s Disease) test battery. Patients were also characterized by assessing their patient files for demographic and clinical data.

**Results:**

The patients’ cognitive subdomains did not improve according to CERAD in their z-scores before and after immunotherapy. We noted a non-significant trend toward an improvement in semantic fluency and verbal memory consolidation. Patients did not do worse in 4 of 12 (33%) cognitive subdomains in the CERAD test battery. Furthermore, mood dysfunction lessened as a non-significant trend in specific psychopathological features such as reduced affective symptoms, loss of drive, and ruminations. Affective symptoms, loss of drive and ruminations were reduced by 43% after immunotherapy.

**Discussion:**

Our small pilot study revealed no relevant alleviation of cognitive dysfunction in patients with neural autoantibodies. However, mood dysfunction became less obvious in specific functions concerning affect, drive, and rumination. However, we do not know whether methylprednisolone affects mood dysfunction, as some patients were taking antidepressant drugs at the same time. Our results might indicate that methylprednisolone immunotherapy is associated with impeding the progression of cognitive dysfunction and reducing mood dysfunction. Further large-scale, placebo-controlled studies in a more homogeneous patient population presenting a uniform pattern of neural autoantibodies should be undertaken.

## Introduction

Cognitive impairment can be associated with anti-neural autoantibodies (Hansen, 2021). Such autoantibodies can be divided into two groups targeting membrane-surface and intracellular antigens. Anti-neural autoantibody-associated cognitive impairment can be part of an autoimmune dementia together with brain inflammation and the patient’s response to immunotherapy (Flanagan et al., 2010), or with autoimmune encephalitis if it coincides with other clinical features such as seizures or psychiatric features (Graus et al., 2016; Abboud et al., 2021a). However, an autoimmune dementia is unlikely if immunotherapy fails to lead to improved cognitive function. The basis of autoimmune dementia is an encephalopathy found to be associated with autoantibodies against membrane-surface antigens (Flanagan et al., 2010; Banks et al., 2021). If autoantibodies against intracellular antigens are detected, a paraneoplastic encephalopathy might often be the origin of autoimmune dementia (Banks et al., 2021). Furthermore, if magnetic resonance imaging fails to reveal a neurodegenerative pattern, an autoimmune origin is likely if other additional criteria such as CSF pleocytosis are fulfilled (Flanagan et al., 2010). The diagnosis of a probable autoimmune encephalitis is made if certain criteria are met such as indices for inflammation in MRI or CSF (Graus et al., 2016; Abboud et al., 2021a). However, several cases in memory clinics do not met the criteria for both disease entities. However, specific autoantibody testing has demonstrated neural autoantibodies in conjunction with signs of neurodegeneration in brain MRI and CSF. There are no therapy guidelines yet for such patients when an autoimmune genesis is likely, but not verifiable through the aforementioned criteria. This is the therapeutic dilemma that led us to perform this pilot study. A large cohort series with autoantibody-associated psychiatric syndromes including cognitive impairment (Endres et al., 2020) indicated that immunotherapy in such cases is highly beneficial. The aim of our retrospective case series study was to assess whether neuropsychological and psychopathological measures improve if immunotherapy with corticosteroids as first-line therapy for autoimmune encephalitis (Abboud et al., 2021b) is applied as an individual health trial.

## Materials and methods

We included seven patients presenting anti-neural autoantibody-associated cognitive impairment in this retrospective case series investigation. The patients were informed about potential effects and side effects of high-dosage methylprednisolone as a once-monthly intravenous therapy applied over 6 cycles. Cognition was evaluated *via* the (Consortium to Establish a Registry for Alzheimer’s Disease) CERAD-Plus testing battery before and 3–6 months after starting immunotherapy. We divided patients into those with a mild neurocognitive impairment (termed MCI) and a major neurocognitive impairment termed as dementia according to the fifth version of the Diagnostic and Statistical Manual of Mental Disorders (DSM-5) (American Psychiatric Association, 2013). Psychopathology was evaluated at the start and at follow-up at the end of the immunotherapy application according to patient files. We used the Manual for the Assessment and Documentation of Psychopathology in Psychiatry (AMDP) (Broome et al., 2017). Psychopathology was assessed applying a dichotomous scale ranging from present (means = 1) or not present (means = 0). 1.5 T magnetic resonance imaging (MRI) were done in the Department of Neuroradiology, University Medical Center Göttingen or off-site at neuroradiologic centers in Göttingen. Autoantibodies were screened in the Euroimmun Laboratory in Lübeck, Germany. We measured the following autoantibodies in serum and CSF biomaterial probes: (1) anti-α-amino-3-hydroxy-5-methyl-4-isoxazolepropionic acid receptors 1/2 (anti-AMPAR1/2), (2) anti-amphiphysin, (3) anti-aquaporin 4, (4) anti-contactin associated protein 2 (CASPR2), (5) anti-CV2, (6) anti-dipeptidyl-peptidase-like 6 protein (DPPX), (7) anti-flotillin 1/2, (8) anti-gamma aminobutyric acid B1/2 receptor (GABAB1/2R), (9) anti-glutamic acid decarboxylase (GAD65), (10) anti-glial fibrillary acid protein (GFAP), (11) anti-HuD, (12) anti-leucin rich glioma inactivated protein 1 (LGI1), (13) anti-Ma1/Ma2, (14) anti-neurexin 3alpha, (15) anti-N-methyl-D-aspartate receptor (NMDAR), (16) anti-Ri, (17) anti-SOX1, (18) anti-Tr/DNER, (19) anti-Yo, and (20) anti-Zic4 antibodies. Molecular biomarkers of neurodegeneration and amyloidopathy were determined in the Neurochemistry Laboratory in the Department of Neurology, University Medical Center Göttingen. As normative values we relied on these cut-offs: the molecular biomarker level was non-pathological if (i) total tau protein (t-tau) < 450 pg/ml, (ii) phosphorylated tau protein 181 (p-tau181) < 61 pg/ml, (iii) ß-amyloid 42 (Aß42) > 450 pg/ml, and (iv) the ratio between Aß42/Aß40 × 10 > 0.5. This study concurred with the current version of the Declaration of Helsinki and was approved by our local ethics committee. Our data were subjected to a Shapiro–Wilk analysis for normal distribution. The Mann–Whitney *U* test was run to compare not normally distributed neuropsychological data pre- and post-therapy. Furthermore, to assess the difference between the frequency of psychopathological items before and after therapy, Fisher’s exact test was used. A *p*-level of *p* < 0.05 was considered as significant.

## Results

### Clinical phenotypic characteristics of patients

**FIGURE 1:**
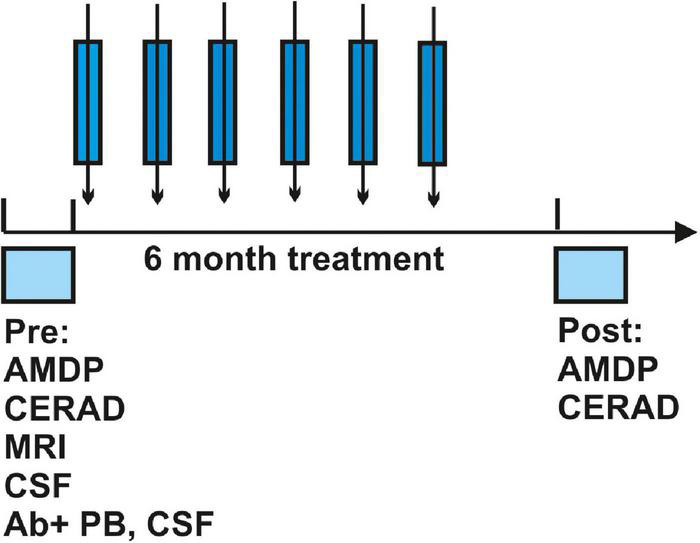
Protocol of the study. The time course of diagnosis, time to treatment, immunotherapeutic treatment regimen as well as post-evaluation after immunotherapy is depicted. Abbreviations: Ab, antibody; AMDP, Manual for the Assessment and Documentation of Psychopathology in Psychiatry; CERAD, Consortium to Establish a Registry for Alzheimer’s Disease; CSF, cerebrospinal fluid; MRI, magnetic resonance imaging; PB, peripheral blood.

Seven patients presenting diverse neural autoantibodies in serum (*n* = 2 neuropil antibodies, *n* = 2 IgLON5 antibodies, *n* = 1 GFAP antibodies, *n* = 1 flotilin 1/2 antibodies, and *n* = 1 neurexin 3alpha antibodies) were identified who exhibited a cognitive impairment (*n* = 3 with dementia, *n* = 4 with MCI). No specific or unspecific CSF autoantibodies were detected in patients. They had a mean age of 66 ± 7 years; the disease had manifested on average 3 years beforehand at age 63 ± 6 years (Table 1). Other laboratory characteristics such as molecular biomarker levels are shown in Table 1. Total tau protein, p-tau 181, Aß42, and the Aß42/40 ratio showed levels in the normative range. One patient only revealed an elevated cell count, three presented a leaky blood-brain barrier. No patient had undergone intrathecal IgG synthesis before starting immunotherapy with methylprednisolone. All patients received six cycles of immunotherapy with methylprednisolone over 6 months (Figure 1). The time to treatment was brief, encompassing in mean 1.28 ± 0.75 months after diagnosis, whereas the time of therapy evaluation lasted a mean 1.64 ± 1.59 months after treatment (Table 1). Predictors of a good immunotherapeutic response according to the Flanagan et al. (2010) study were present, such as brief latency to treatment, but we did not often discern predictors of a good immunotherapeutic response such as a tremor, a dynamic course, and subacute onset (Table 1). Our MRI data revealed no classical neurodegenerative-disease pattern such as Alzheimer’s disease with hippocampal atrophy (Table 1). The clinical presentation is not characterized by a subacute onset or mainly dynamic time course. Furthermore, most of these patients present no cancer history or history of cancer in their family, no dementia in their family, or even a history of autoantibodies (Table 1).

**TABLE 1:** Demographic and clinical patient characteristics.

Demographic parameter	
Sex (female)	7 (3)
Age y	66.1 ± 6.9
Age of onset y	63.3 ± 5.7
Early onset (<65 years), *n*	3/7 (42.9%)
**CSF**	
Cell count (<5 μg/L)	2 ± 2.9
Albumin mg/L	369.4 ± 139
Tau protein (<450 pg/ml)	341 ± 118
P Tau protein 181 (<61 pg/ml)	59.7 ± 12
Aß42 (>450 pg/ml)	1012 ± 450
Aß40	11068 ± 2105
Ratio Aß42/40 ’ 10 (>0.5)	0.93 ± 0.45
**Brain MRI**	
Generalized atrophy	1/7 (14%)
Focal atrophy	4/7 (57%)
Hippocampal atrophy	0/7 (0%)
Contrast enhancement	0/7 (0%)
Vascular lesions	5/7 (71%)
Antidepressant drugs	4/7 (57%)
**Clinical parameter**	
Fluctuations of cognitive impairment	1/7 (14%)
Dynamic time course of symptoms	2/7 (28%)
Tremor	0/7 (0%)
Brief time latency to treatment	6/7 (86%)
Subacute clinical presentation	0/7 (0%)
History of cancer	1/7 (14%)
Family history of cancer	1/7 (14%)
Family history of dementia	0/7 (0%)
Family history of neural autoantibodies	0/7 (0%)
Time between diagnosis and treatment month	1.28 ± 0.75
Time between treatment and evaluation month	1.64 ± 1.59

Abbreviation: CSF, cerebrospinal fluid; IgA, immunoglobulin A; IgG, immunoglobulin G; IgM, immunoglobulin M; MRI, magnetic resonance imaging; P Tau Protein 181, phosphorylated tau protein 181; TMT, Trail Making Test; y, years. The values are depicted as mean ± standard deviation. For laboratory data normal ranges are shown in brackets.

### Cognitive data

Neuropsychological testing demonstrated no improvement or deterioration in cognitive dysfunction expressed as the z-score (see Table 2A) prior to and after immunotherapy. The follow-up neuropsychological investigation was done 8.7 ± 2.5 months after the initial work-up. Patients did not do worse in 4 of 12 (33%) cognitive domains in the CERAD test battery. We observed a mean improvement in z-score (positive z-score difference) after methylprednisolone therapy in the subdomains semantic fluency, figure copying, and verbal memory consolidation (list recall savings) (Table 2A). However, most of the subdomain functions worsened after methylprednisolone, i.e., naming capacity, global level of cognition (MMSE), verbal learning (list learning) and verbal memory recall (list recall) and recognition (list recognition), figure recall, phonematic fluency, and cognitive flexibility (TMT part B).

**TABLE 2A:** Neuropsychological and psychometric data in patients with cognitive dysfunction.

Cognition	Pre z-score	Post z-score	Difference z-score
Semantic fluency	−2.21 ± 1.32	−1.73 ± 1.89	0.45 ± 0.62
Boston naming test	−0.95 ± 2.55	−1.38 ± 2.36	−0.43 ± 0.76
MMSE	−2.5 ± 1.03	−3 ± 2.09	−0.50 ± 0.61
List learning	−1.96 ± 1.43	−2.8 ± 1.85	−0.83 ± 0.41
List recall	−1.78 ± 1.11	−1.96 ± 1.36	−0.18 ± 0.80
List recall (savings)	−1.8 ± 1.14	−1.25 ± 2.02	0.55 ± 0.57
List recognition	−1.73 ± 0.66	−1.9 ± 2.19	−0.16 ± 0.86
Figure copy	0.06 ± 1.62	0.11 ± 0.86	0.05 ± 0.94
Figure recall	−1.56 ± 1.68	−2.4 ± 1.31	−0.33 ± 0.34
Phonematic fluency	−0.9 ± 1.39	−1.38 ± 1.64	−0.48 ± 0.59
TMT part A	−0.4 ± 0.63	−0.23 ± 0.86	0.1 ± 0.74
TMT part B	0.48 ± 1.21	−0.16 ± 0.93	−1.3 ± 0.63
**Psychopathology**			
Orientation disturbances	1/7 (14%)	1/7 (14%)	0/7 (0%)
Memory disturbances	7/7 (100%)	7/7 (100%)	0/7 (0%)
Formal thought disorder	6/7 (86%)	3/7 (43%)	3/7 (−43%)
Mood dysfunction	6/7 (86%)	3/7 (43%)	3/7 (−43%)
Loss of drive	6/7 (86%)	3/7 (43%)	3/7 (−43%)
Circadian disturbances	2/7 (28%)	3/7 (43%)	1/7 (+14%)

### Psychopathological data

Our patients’ main psychopathological features did not change before or after immunotherapy with methylprednisolone. Formal thought disorder, affect, and psychomotor drive were less affected after immunotherapy compared to the state prior to immunotherapy as a non-significant trend (Table 2A). Formal thought disorder, affective symptoms and the loss of drive were reduced by 43% after immunotherapy (Table 2A). Interestingly the affect symptoms and loss of drive were even more reduced (−50%) in those patients in the dementia group, but not in MCI patients (Tables 2B,C). Formal thought disorder was diminished in both MCI and dementia patients (Tables 2B,C). The reduced occurrence of affective disturbance, loss of drive and formal thought disorder was noted in one patient not undergoing antidepressant drug treatment, but also in another taking antidepressants. Four of our patients were taking antidepressants (57%), but depressive symptoms only resolved in one patient (25%). Depressive symptoms (affective symptoms, loss of drive, rumination as formal thought disorder) became weaker in 2 of 5 (40%).

**TABLE 2B:** Neuropsychological and psychometric data in patients with dementia.

Cognition	Pre z-score	Post z-score	Difference z-score
Semantic fluency	−1.95 ± 1.86	−1.97 ± 1.63	−0.025 ± 0.98
Boston naming test	−1.43 ± 3.09	−1.72 ± 2.71	−0.3 ± 0.88
MMSE	−2.72 ± 1.05	−3.48 ± 2.43	−0.75 ± 0.6
List learning	−2.2 ± 1.45	−3.3 ± 1.84	−1.1 ± 0.37
List recall	−2.1 ± 1.03	−2.3 ± 1.52	−0.17 ± 0.85
List recall (savings)	−2.3 ± 1.2	−1.8 ± 2.24	0.42 ± 0.75
List recognition	−1.7 ± 0.49	−2.27 ± 2.21	−0.55 ± 0.65
Figure copy	−0.27 ± 1.98	0.25 ± 0.89	0.52 ± 0.65
Figure recall	−1.77 ± 1.93	−2.4 ± 1.2	0.33 ± 0.56
Phonematic fluency	−1.03 ± 1.26	−1.6 ± 1.69	−1.02 ± 1.26
TMT part A	−0.63 ± 0.54	−0.47 ± 0.92	0.15 ± 0.79
TMT part B	−0.3 ± 0.99	−1.9 ± 0.95	−
**Psychopathology**			
Orientation disturbances	1/4 (25%)	1/4 (25%)	0%
Memory disturbances	4/4 (100%)	4/4 (100%)	0%
Formal thought disorder	3/4 (75%)	1/4 (25%)	−50%
Mood dysfunction	3/4 (75%)	1/4 (25%)	−50%
Loss of drive	3/4 (75%)	1/4 (25%)	−50%
Circadian disturbances	1/4 (25%)	1/4 (25%)	0%

**TABLE 2C:** Neuropsychological and psychometric data in patients with mild cognitive impairment.

Cognition	Pre z-score	Post z-score	Difference z-score
Semantic fluency	−2.6 ± 0.83	−1.25 ± 2.36	1.1 ± 0.66
Boston naming test	0.0 ± 1.13	−0.7 ± 2.12	−0.7 ± 0.73
MMSE	−2.05 ± 1.2	−2.05 ± 1.2	0
List learning	−1.5 ± 1.83	−1.75 ± 1.9	−0.25 ± 0.9
List recall	−1.1 ± 1.27	−0.6 ± 1.6	0.2 ± 0.87
List recall (savings)	−0.95 ± 0.21	−0.15 ± 1.34	0.8 ± 0.55
List recognition	−1.75 ± 1.2	−1.15 ± 2.75	0.6 ± 0.81
Figure copy	0.75 ± 0.21	−0.15 ± 1.06	−0.9 ± 0.43
Figure recall	−0.7 ± 0.49	−	−
Phonematic fluency	−0.65 ± 2.19	−1 ± 2.12	0.35 ± 0.88
TMT part A	0.5 ± 0.35	0.25 ± 0.76	−
TMT part B	1.25 ± 1.44	0.7 ± 0.72	−0.55 ± 0.74
**Psychopathology**			
Orientation disturbances	0/3 (0%)	0/3 (0%)	0%
Memory disturbances	3/3 (100%)	3/3 (100%)	0%
Formal thought disorder	3/3 (100%)	2/3 (66%)	−34%
Mood dysfunction	2/3 (66%)	2/3 (66%)	0%
Loss of drive	2/3 (66%)	2/3 (66%)	0%
Circadian disturbances	1/3 (33%)	2/3 (66%)	+33%

MMSE, Mini Mental Status Examination; TMTA, Trail Making Test part A; TMTB, Trail Making Test part B.

## Discussion

Our findings suggest that immunotherapy with methylprednisolone does not improve cognition associated with anti-neural autoantibodies. However, as we observed no relevant deterioration in cognitive impairment either, we believe that immunotherapy might be beneficial to slow down the progression of cognitive loss. The effects of reduced depressive symptoms accompanied by less loss of drive, fewer affective symptoms, and a weaker formal thought disorder imply that immunotherapy or antidepressant therapy might have an effect. Our case series approach does not enable us to clarify whether it is the drug therapy (as either immunotherapy or antidepressants) that is effective in reducing depressive symptoms. Antidepressants were being taken by 57% of patients, but helped only 25% of them. Thus, other factors like immunotherapy might be also effective such as antidepressant drugs. If a neuropsychiatric syndrome involving cognitive impairment (and depressive symptoms in some patients) is assumed, it is tempting to link immunotherapy’s mild beneficial effect to the neuropsychiatric symptoms. A small cohort study of ours showed that mixed immunotherapy including methylprednisolone therapy was effective in improving mood dysfunction in epilepsy patients presenting GAD65 autoantibodies rather than paraneoplastic autoantibodies (Hansen et al., 2016). Flanagan et al. (2010) showed furthermore that 15/46 (33%) of patients with suspected autoimmune dementia and additional depressed mood were responsive to corticosteroids, whereas 10/26 (38%) of patients revealed no clear effect. There is additional evidence from small patient cohorts and case reports that both unipolar and bipolar depression are associated with neural autoantibodies like NMDAR autoantibodies, and that they respond to corticosteroids (Choe et al., 2013; Restrepo-Martínez et al., 2020). In other words, corticosteroids can be tricky within a psychiatric patient population on the one hand, as psychiatric symptoms may get worse, but on the other hand a beneficial effect is likely; to prove this, large cohort studies are necessary in patients with cognitive impairment and a depressive syndrome associated with anti-neural autoantibodies. However, the absence of any alleviation of cognitive dysfunction is surprising, as good responsivity of 64% in patients with autoimmune dementia was seen in another cohort of Flanagan et al. (2010). These inconsistent results arise from different cohorts presenting a diverse and heterogeneous spectrum of associated anti-neural autoantibodies. These findings might also be influenced by the inconsistent and sometimes dynamic development of symptoms in autoimmune dementia or cognitive impairment. Furthermore, the time interval of our evaluations was about 1.6 months after the last therapeutic intervention, so that our findings also could also reflect the natural development and may not necessarily be attributable to the treatment itself.

### Limitations

Our small patient sample is a major limitation, as no clinical clues can be drawn from this investigation. However, this pilot data suggests a direction, namely, that immunotherapy does not tend to make cognition deteriorate. However, as we enrolled no control cohort, we cannot claim that methylprednisolone has an effect, as we cannot know how the disease course would proceed when comparing in healthy controls to disease controls undergoing this type of immunotherapy. A study by Flanagan et al. (2010) examining antibody-mediated dementia (autoimmune dementia) demonstrated that a positive response to immunotherapy was predicted by a subacute onset, dynamic course, tremor, and briefer time latency to treatment. Another limitation is our subgroup analysis of MCI and dementia patients. Dementia patients seem to have less formal-thought disorder, fewer affective symptoms, and less loss of drive. However, these subgroups are too small to draw conclusion about cognitive-impairment subgroups, of thus more research is needed to confirm these findings.

## Conclusion

These study results of ours encourage us to conduct larger cohort studies to investigate the efficacy of methylprednisolone in autoantibody-associated cognitive decline. The brief latency to treatment was the only of our cohort’s parameters in our that according to Flanagan et al. (2010) predicted the good outcome of reduced affective symptoms as a dynamic course was seldom apparent and tremor and a subacute course were absent. The results of our subgroup analysis suggest that dementia patients might even benefit more than MCI patients regarding the reduction in affective symptoms, although the small sample size limits this assumption. We will investigate whether other symptoms besides cognitive impairment, such as depressive symptoms, are also modulated by corticosteroids in larger-scale cohort studies.

## Data availability statement

The raw data supporting the conclusions of this article will be made available by the corresponding author, without undue reservation.

## Ethics statement

The study involving human participants was reviewed and approved by Ethics committee of University Medical Center Göttingen. Written informed consent for participation was not required for this study in accordance with the national legislation and the institutional requirements.

## Author contributions

NH wrote the manuscript. SH, KR, BM, and DF had revised the manuscript for important intellectual content. All authors contributed to the article and approved the submitted version.
